# Effectiveness of a family–school–community collaborative physical activity intervention

**DOI:** 10.3389/fpubh.2026.1767961

**Published:** 2026-02-27

**Authors:** Lin Kong, Xinyu Chen, Binping Gong, Xiqian Zhang, Mingming Guo

**Affiliations:** 1 School of Physical Education, Guangdong University of Education, Guang Zhou, China; 2 Department of Physical Education, College of Education for the Future, Beijing Normal University, Zhu Hai, China; 3 College of Physical Education and Sport, Beijing Normal University, Beijing, China

**Keywords:** exercise benefits, exercise cognition, family–school–community collaboration, physical activity, primary school students, school-based intervention

## Abstract

**Background:**

Family–school–community collaboration is increasingly recognized as a strategy to promote physical activity (PA) in youth, yet evidence in primary school settings—particularly for exercise-related cognition—remains limited.

**Objective:**

To evaluate a family–school–community collaborative PA intervention on PA and exercise cognition in primary school students, and to examine associations between PA and exercise cognition.

**Methods:**

In this quasi-experimental, one-academic-year study, 515 fifth-grade students (10–11 years) from a public primary school in Guangdong, China, were allocated to an intervention group (*n* = 255) or a control group (*n* = 260). The intervention group received a structured family–school–community collaborative PA program, while controls received usual physical education. PA was assessed using PAQ-CN; exercise cognition (benefits/barriers) was measured using EBBS-CN. Intervention effects were estimated using class-clustered ANCOVA adjusting for baseline outcome values and sex; exploratory associations were examined using class-clustered regression models.

**Results:**

PA increased from pre- to post-intervention in both groups. In adjusted class-clustered ANCOVA models, the intervention was associated with higher exercise cognition (*β* = 3.94, 95% CI: 1.29–6.60; *p* = 0.003; Hedges’ *g* = 0.26) and higher perceived exercise benefits (*β* = 3.46, 95% CI: 1.03–5.89; *p* = 0.008; Hedges’ *g* = 0.24). The adjusted between-group effect on PA was not statistically significant (*β* = 0.11, 95% CI: −0.15–0.36; *p* = 0.418; Hedges’ *g* = 0.18), nor was the effect on exercise barriers (*β* = 0.55, 95% CI: −0.60–1.70; *p* = 0.354; Hedges’ *g* = 0.08). In exploratory class-clustered regression models (with baseline PA adjustment), post-intervention PA was positively associated with exercise cognition and perceived exercise benefits (both *p* ≤ 0.001), but not with perceived exercise barriers (*p* = 0.453).

**Conclusion:**

In this quasi-experimental, single-school study, the intervention was associated with improvements in exercise cognition and perceived benefits, while incremental between-group gains in PA were modest and not statistically robust after accounting for class clustering. These findings support multi-contextual approaches and underscore the potential value of strengthening positive exercise perceptions within coordinated PA promotion efforts.

## 1 Introduction

Physical activity (PA) refers to any bodily movement produced by skeletal muscles that results in energy expenditure, including activities undertaken at work, during transportation, at home, or in leisure-time settings (1). Among children and adolescents, higher levels of PA—particularly moderate-to-vigorous physical activity (MVPA)—have been associated with a range of favorable health outcomes, such as better cardiorespiratory and muscular fitness, healthier cardiometabolic profiles, and improved bone health (2). Beyond physical health, growing evidence links PA to better mental health in young people, including lower depressive and anxiety symptoms and higher psychological wellbeing (3, 4). Accordingly, promoting PA during school age is widely recognized as a key strategy to support both physical and psychological development (5).

According to the World Health Organization (WHO) 2020 Guidelines on Physical Activity and Sedentary Behaviour, children and adolescents aged 5–17 years should accumulate an average of at least 60 min per day of MVPA across the week and include muscle- and bone-strengthening activities on at least 3 days per week (6, 7). Nevertheless, global surveillance indicates that more than 80% of adolescents do not meet these recommendations, and this pattern co-occurs with increasing sedentary time and rising obesity prevalence (8–10). In China, nationally representative surveys similarly suggest that only about one-third of school-aged children and adolescents achieve the recommended ≥60 min/day of MVPA, and over 20% are overweight or obese (11, 12). These patterns underscore the need for effective PA promotion during school age, yet interventions delivered in a single setting often face practical constraints. Specifically, school-only approaches may be constrained by fixed timetables and academic demands that limit discretionary MVPA beyond scheduled PE (13), whereas family-only strategies can be hindered by parents’ time constraints and unequal access to safe and affordable activity opportunities (14). In addition, community resources (e.g., facilities, organized events, and volunteer support) are not always readily mobilized without coordination across stakeholders (15). These contextual constraints suggest that isolated single-setting interventions may yield limited or unsustained effects (16), thereby motivating a coordinated family–school–community approach that aligns messages, opportunities, and social support across settings (16).

**Figure 1:**
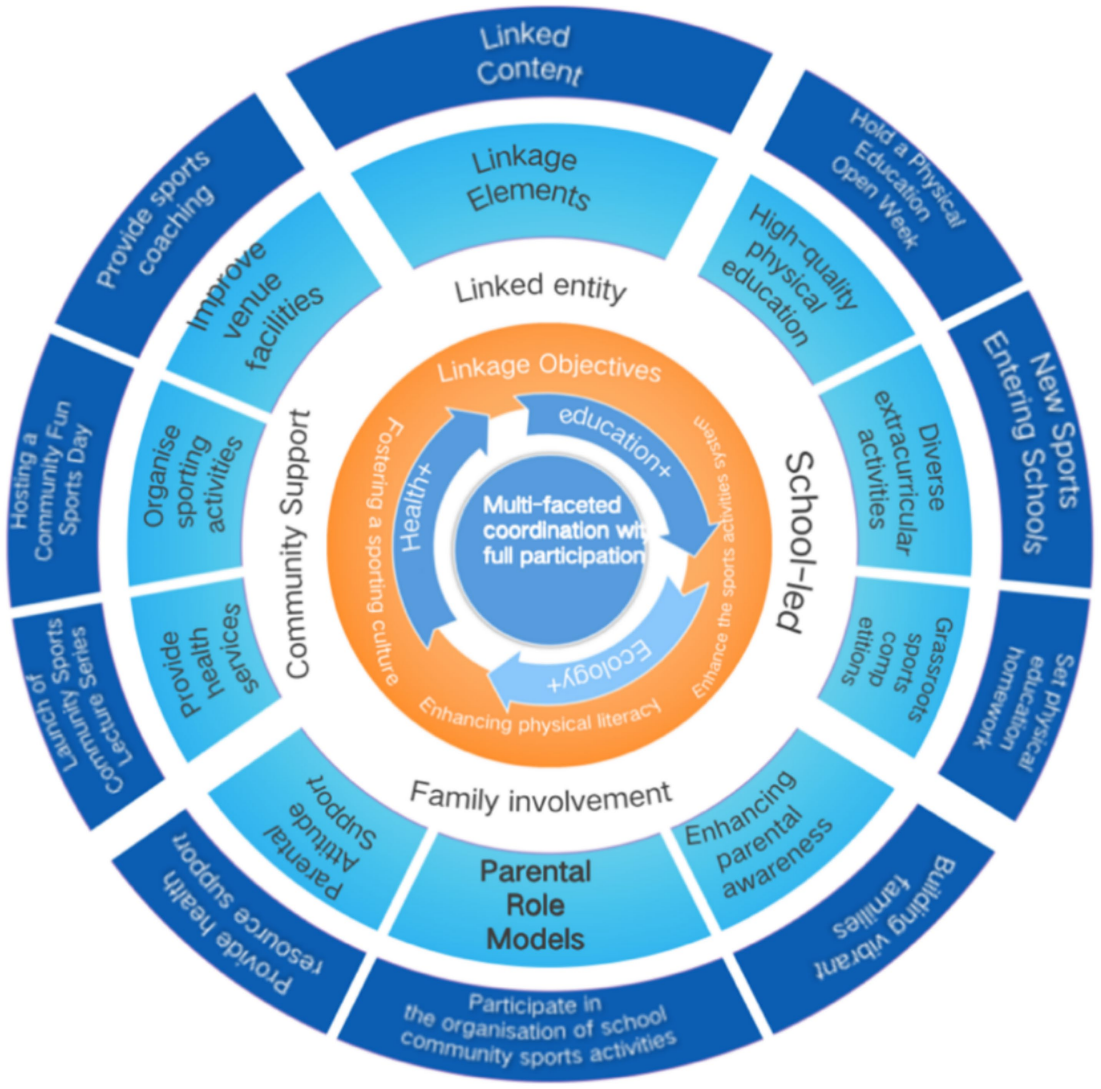
Logic model (theory of change) for the multi-dimensional family–school–community collaborative intervention.

In this context, multilevel PA interventions grounded in the Social Ecological Model and CSPAP frameworks have been increasingly advocated for promoting PA in school-aged youth (17, 18). Consistent with these frameworks, we conceptualize family–school–community collaboration as a coordinated multisetting approach; specifically, we define a family–school–community collaborative intervention as a coordinated, multi-setting approach in which schools provide structured PA opportunities and supportive norms, families reinforce PA through home-based practice and parental support, and community partners expand access to resources and activity opportunities, with aligned goals and communication across stakeholders (19, 20). In the present study, the intervention components were designed to operate across these settings and stakeholders (Table 1; Figure 1). To make the hypothesized pathway explicit, we developed a logic model (theory of change) linking multi-setting inputs to expected changes in children’s PA and related determinants (Figure 1).

**Table 1:** Design of physical activity intervention content based on family–school–community collaboration.

Linking entities	Implementation content	Implementation frequency	Duration
School–family	After-school sports activities	Once a day	1 h per session
School–family	Sports homework	5 times a week	30 min per session
School–family	Active family development	Evaluated monthly	Ongoing activities
School–family	Weekend parent–child activities	Once a month per experimental class	2 h per session
School–family	Sports class open day	1–2 times per semester per experimental class	1 class per session
School–family–community	Community sports carnival	Fun sports activities with themes like Learn from Lei Feng, Labor Day, National Day, etc.	2 h per session
School–family–community	Sports volunteer team	Volunteer service by teachers, parents, and community workers	Ongoing participation in sports activity guidance, organization, management, decision-making
School–family–community	Health education activities	Lectures, health science popularization, sports skill guidance, etc.	Once a month

Prior research suggests that coordinated, multi-setting approaches can improve PA-related outcomes in youth, but effects are heterogeneous and may be constrained by implementation challenges and limited evidence on sustained, academic-year impacts (16, 18–21). Moreover, many studies prioritize behavioral endpoints while providing limited concurrent evidence on theoretically proximal cognitive constructs and implementation fidelity, which constrains interpretation of “what works, for whom, and through which pathways,” particularly in context-specific school settings. Given that PA promotion depends on both multi-setting opportunities/support and children’s belief-based appraisals, we specified PA level as the primary outcome and “exercise cognition” as a theoretically relevant secondary outcome (22, 23). Exercise cognition refers to perceived exercise benefits and barriers and was operationalized using the EBBS total score, with benefits and barriers subscales examined to support interpretability (22, 24). Accordingly, this study aimed to evaluate, using a one-academic-year quasi-experimental design, whether the family–school–community collaborative intervention was associated with changes in PA and exercise cognition among primary school students, and to examine their association.

## 2 Methods

### 2.1 Research design and participants

**Figure 2:**
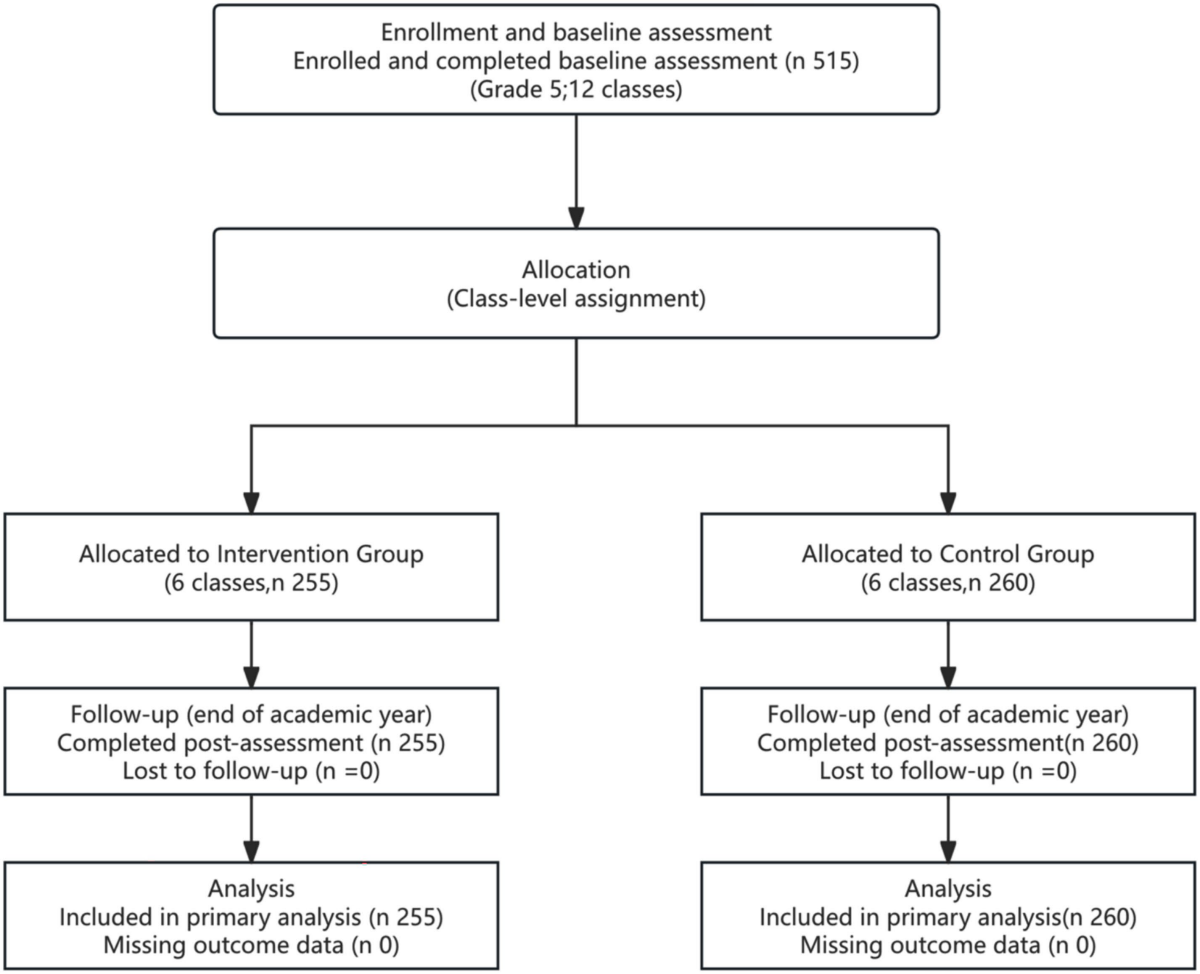
Participant flow diagram. A total of 515 grade 5 students (12 classes) were enrolled and completed the baseline assessment. Classes were assigned at the class level to the intervention group (6 classes, *n* = 255) or the control group (6 classes, *n* = 260). All students completed the post-intervention assessment at the end of the academic year; no participants were lost to follow-up and no outcome data were missing for the primary analyses (total analyzed: *N* = 515).

This study adopted a quasi-experimental design, conducted over one academic year in a public primary school in Guangdong Province. All Grade 5 students (ages 10–11 years) from the school’s 12 intact classes were recruited (*N* = 515). The 12 natural classes were assigned at the class level to an intervention group (6 classes, 255 students; 55.29% male) or a control group (6 classes, 260 students; 49.62% male). Baseline sex distribution did not differ significantly between groups (Table 2). A detailed participant flow diagram, including enrollment, class-level allocation, follow-up, and analysis, is provided in Figure 2.

**Table 2:** Baseline sex distribution by group (*N* = 515).

Group	Male, *n* (%)	Female, *n* (%)	Total, *n*
Intervention group	141 (55.29%)	114 (44.71%)	255
Control group	129 (49.62%)	131 (50.38%)	260
Total	270 (52.43%)	245 (47.57%)	515

Values are *n* (%). Group differences in sex distribution were assessed using a *χ*^2^ test (two-sided).

Given the single-school, same-grade cohort, we expected relatively limited between-group heterogeneity in background characteristics; however, household socio-demographic variables (e.g., parental education, socioeconomic status, and residence) were not collected and therefore could not be compared formally.

This same-school implementation also introduced a risk of contamination (i.e., spillover of intervention content to control classes through student interactions). To mitigate this risk, the intervention was delivered at the class level, and intervention materials and guidance (e.g., parent workshops and sports homework instructions) were provided only to intervention classes and their guardians, while the control classes continued usual physical education without access to the intervention resources; nevertheless, complete separation between classes could not be ensured.

Regarding retention and data completeness, all enrolled students completed both baseline and post-intervention assessments (no loss to follow-up), and outcome data were complete for the primary analyses. Prior to the study, written guardian consent and student assent/informed consent were obtained.

### 2.2 Procedure

This study was approved by the Ethics Committee of East China Normal University. Participants were recruited with the assistance of the participating school from Grade 5 intact classes, and eligibility was confirmed by the research team in coordination with school staff. Written guardian consent and student assent/informed consent were obtained prior to data collection.

PA levels, exercise barriers, exercise benefits, and overall exercise cognition were assessed in both groups at two time points: baseline (late September, pre-intervention) and post-intervention (late May, end of the academic year). To reduce potential variation due to scheduling, assessments were administered in classrooms during regular school hours by trained PE teachers using standardized, scripted instructions provided by the research team. All questionnaires were completed individually by students under teacher supervision.

### 2.3 Intervention and fidelity

The intervention was implemented in a public primary school in Guangdong Province. Both groups followed the national compulsory education PE curriculum; the intervention group additionally received a structured family–school–community collaborative program (regular parent lectures/workshops, structured school break-time PA activities, and family/community sports events; see Table 1 and Figure 1) alongside usual PE, whereas the control group continued usual PE only (5 lessons/week, 40 min/lesson).

Intervention delivery was school-led and primarily teacher-delivered. School-based components were delivered by PE teachers; family components (e.g., parent workshops and homework guidance) were delivered by PE teachers with support from the research team; community events were organized by the school (teachers/volunteers). To promote consistency across the six intervention classes, core materials, task templates, and weekly schedules were standardized, and implementation was reviewed in periodic coordination meetings with PE and class teachers.

Intervention fidelity and participation were monitored using routine school records and verification procedures. For sports homework (5 times/week), PE teachers issued standardized weekly tasks, and completion was documented via class-level homework logs collected by class teachers, with guardian confirmation where applicable; teachers periodically reviewed logs and issued reminders through class communication channels. For community events, attendance was recorded using on-site sign-in sheets/registration records, and class-level participation lists were compiled. Across the academic year, the research team held regular meetings with PE/class teachers to verify delivery schedules, address implementation issues, and minimize contamination by restricting intervention materials and guidance to intervention classes and their guardians. Participation was verified at the class level; however, we did not compute a student-level adherence (“dose”) index for formal modeling.

Outcome assessments were administered using the same tools and scoring standards in both groups at baseline and post-intervention by a trained team.

### 2.4 Tools and measurement

*PA Questionnaire for Children—Chinese version (PAQ-CN).* PA was assessed using the Chinese version of the Physical Activity Questionnaire for Children (PAQ-CN), adapted from the original PAQ developed by Kowalski et al. (25). The PAQ/PAQ-CN is designed for children and adolescents aged 7–18 years and has been widely used to assess general PA levels in youth (11, 26, 27). The PAQ-CN is a self-administered, 7-day recall questionnaire with 10 items; nine items capture PA participation over the previous week (e.g., activity type and frequency) and are scored on a 1–5 scale. The PA score is calculated as the mean of the nine activity items; scores are categorized as low (≤2), moderate (2–3), and high (>3) (25). The PAQ-CN has demonstrated acceptable reliability in Chinese samples (*α* = 0.821) and has been used in prior Chinese pediatric studies (11, 28). Given participants’ age (10–11 years), questionnaires were completed in class under teacher supervision using standardized instructions. As a self-report measure, PAQ-CN may be subject to recall and social desirability bias.

*Exercise Benefits/Barriers Scale—Chinese version (EBBS-CN)*. Exercise-related cognition was assessed using the Chinese version of the Exercise Benefits/Barriers Scale (EBBS-CN), originally developed by Sechrist et al. (29). The EBBS comprises two dimensions—perceived exercise benefits and perceived exercise barriers—with five benefits subdomains (life enhancement, physical performance, psychological outlook, social interaction, and preventive health) and four barriers subdomains (exercise environment, time management, physical exertion, and family support) (29). Higher scores indicate higher perceived benefits and/or higher perceived barriers. The EBBS-CN has shown high internal consistency in Chinese samples (*α* = 0.961) ((30)).

### 2.5 Data analysis

Descriptive statistics are reported as mean (SD) by group (intervention vs. control) and time (pre vs. post). Primary intervention effects were estimated using ANCOVA, with each post-intervention outcome modeled as Outcome_post = *β*₀ + *β*₁(treat) + *β*₂(Outcome_pre) + *β*₃(female) + *ε*. Because students were nested within classes, robust standard errors were clustered at the class level (12 classes). Treatment effects are reported as *β*₁ with 95% CIs, together with standardized effect sizes (Hedges’ *g*, standardized by the pooled baseline SD).

Given the small number of clusters, we conducted small-cluster–robust inference in addition to cluster-robust ANCOVA. First, we performed an exact class-level permutation test by enumerating all allocations of six treated classes among 12 (*C*(12,6) = 924) and computing two-sided permutation *p*-values using *β*₁ as the test statistic. Second, we implemented a wild cluster bootstrap-t procedure with Webb six-point weights to obtain bootstrap-t confidence intervals.

Sensitivity analyses used a class-level difference-in-differences (DID) approach based on class-mean change scores (post–pre). For multiplicity, PA and exercise cognition were specified as co-primary outcomes; exercise benefits and exercise barriers were treated as secondary outcomes, and their p-values were Holm-adjusted. Associations between PA and exercise cognition (and subscales) were examined as exploratory using regression models with class-clustered robust standard errors and baseline adjustment. Baseline intraclass correlation coefficients (ICCs) were estimated for each outcome to quantify class-level clustering. All tests were two-sided with *α* = 0.05, and analyses were performed in Python.

## 3 Results

### 3.1 Descriptive results

Descriptive statistics for all outcomes by group and time are presented in Table 3. Both groups showed increases from pre- to post-intervention in physical activity and exercise cognition, with descriptively larger mean gains in the intervention group for physical activity, exercise cognition, and exercise benefits (Table 3).

**Table 3:** Descriptive statistics of outcomes by group and time (mean ± SD).

Outcome	Intervention group (255)		Control group (260)	
	Pre	Post	Pre	Post
Physical activity	2.02 ± 0.49	2.42 ± 0.57	1.98 ± 0.76	2.31 ± 0.55
Exercise cognition	131.85 ± 12.79	135.30 ± 16.81	129.17 ± 19.23	131.27 ± 14.99
Exercise benefits	88.85 ± 11.28	91.30 ± 14.80	87.40 ± 17.72	87.90 ± 12.41
Exercise barriers	43.00 ± 4.87	44.00 ± 7.65	41.77 ± 9.53	43.37 ± 5.96

Values are M ± SD. M, mean; SD, standard deviation. Physical activity was measured using PAQ-CN (1–5; higher scores indicate higher activity). Exercise cognition (EBBS-CN total), exercise benefits (EBBS-CN benefits subscale), and exercise barriers (EBBS-CN barriers subscale) were measured using EBBS-CN; higher scores indicate higher perceived benefits/barriers and higher overall benefit–barrier appraisal.

### 3.2 Primary outcomes: class-clustered ANCOVA

To account for class-level clustering, intervention effects were estimated using ANCOVA models adjusting for baseline values of each outcome and sex, with robust standard errors clustered at the class level (12 classes; analytic *N* = 515). Results are summarized in Table 4.

**Table 4:** Primary ANCOVA results for intervention effects with class-clustered robust standard errors.

Outcome	Treat effect, *β*	SE (clustered by class)	95% CI	p (clustered)	Hedges g (baseline SD)	N students	N classes
Physical activity	0.106	0.131	−0.151, 0.363	0.419	0.166	515	12
Exercise cognition	3.940	1.302	1.388, 6.492	0.002^**^	0.240	515	12
Exercise benefits	3.460	1.300	0.913, 6.007	0.008^**^	0.232	515	12
Exercise barriers	0.553	0.585	−0.594, 1.699	0.345	0.073	515	12

ANCOVA models were specified as Outcome_post = *β*_0_ + *β*_1_(treat) + *β*_2_(Outcome_pre) + *β*_3_(female) + *ε*. SE, standard error; CI, confidence interval. Robust SEs were clustered at the class level (12 classes). *β* represents the adjusted between-group difference at post-intervention conditional on baseline outcome and sex. Effect sizes are reported as Hedges’ *g* standardized by the pooled baseline SD. Two-sided tests were used. Significance levels: ^*^p < 0.05, ^**^p < 0.01 (two-sided).

For the co-primary outcomes, the intervention was associated with a statistically significant improvement in exercise cognition (*β* = 3.940, 95% CI: 1.388 to 6.492; *p* = 0.002; Hedges’ *g* = 0.240). In contrast, the adjusted between-group difference in physical activity at post-intervention was not statistically significant (*β* = 0.106, 95% CI: −0.151 to 0.363; *p* = 0.419; Hedges’ *g* = 0.166) (Table 4). The standardized effects for both co-primary outcomes were small (g ≈ 0.17–0.24), and estimates are interpreted accordingly.

### 3.3 Secondary outcomes and small-cluster robustness

For secondary outcomes, the intervention was associated with higher exercise benefits (*β* = 3.460, 95% CI: 0.913 to 6.007; *p* = 0.008; Hedges’ *g* = 0.232), while the effect on exercise barriers was not statistically significant (*β* = 0.553, 95% CI: −0.594 to 1.699; *p* = 0.345; Hedges’ *g* = 0.073) (Table 4). Multiplicity control was applied to the secondary outcome family only (exercise benefits and barriers) using the Holm procedure; exercise benefits remained significant after adjustment (p_Holm = 0.0155), whereas exercise barriers did not (Table 5). Similarly, the effect size for exercise benefits was small (Hedges’ *g* ≈ 0.23), suggesting limited practical magnitude despite statistical significance.

**Table 5:** Multiplicity handling (co-primary outcomes and Holm adjustment for secondary outcomes).

Outcome	Role	*p* (raw)	*p* (adjusted)	Adjustment
Physical activity	Co-primary	0.419	–	None (co-primary)
Exercise cognition	Co-primary	0.002^**^	–	None (co-primary)
Exercise benefits	Secondary	0.008^**^	0.016	Holm (secondary family)
Exercise barriers	Secondary	0.345	0.345	Holm (secondary family)

Holm adjustment was applied to the secondary outcome family only (exercise benefits and barriers). Co-primary outcomes (physical activity and exercise cognition) were interpreted without secondary-family adjustment. Two-sided tests were used. Significance levels: ^*^p < 0.05, ^**^p < 0.01 (two-sided).

### 3.4 Robustness and supplementary analyses

Given the small number of clusters (12 classes), we conducted small-cluster–robust inference. Exact class-level permutation tests and wild cluster bootstrap-t confidence intervals (Webb weights) were broadly consistent with the primary class-clustered ANCOVA results, supporting the intervention effects for exercise cognition and exercise benefits, but not for physical activity or exercise barriers (Supplementary Tables S1–S2). Class-level DID using class mean change scores yielded directionally similar but non-significant estimates (Supplementary Table S3). Exploratory association analyses suggested positive associations of exercise cognition/benefits with PA at post and for change scores, and baseline ICCs indicated modest clustering, particularly for PA (ICC = 0.114) (Supplementary Tables S4–S6). Because PA increased from pre to post in both groups (Table 3), interpretation of PA effects emphasizes adjusted between-group estimates rather than within-group change.

## 4 Discussion

This study evaluated a one-academic-year family–school–community PA intervention in primary school students. The intervention was associated with improved exercise cognition and perceived benefits, whereas no clear effects were observed for perceived barriers or PA after accounting for class clustering. Although PA increased in both groups, the incremental between-group difference was small and non-significant, and observed effects were small in standardized magnitude, underscoring that statistical significance does not necessarily imply substantial behavioral impact.

We interpret these findings within a socio-ecological and social-cognitive framework: aligned school opportunities, family reinforcement, and community support may shift benefit–barrier appraisals earlier than measurable changes in PA, particularly under contextual influences and self-report measurement constraints. This pattern is consistent with a “cognition-first” pathway in which attitudinal change may precede detectable behavioral divergence.

### 4.1 Impact of the family–school–community collaborative intervention on primary school students’ physical activity

PA in late childhood often requires coordinated support across multiple settings, because opportunities to be active are shaped not only by school provision but also by family routines and access to community resources (16, 31). In the present study, PA increased from pre- to post-intervention in both groups, with a larger mean increase in the intervention group. However, after adjustment for baseline PA and sex and accounting for class clustering, the incremental between-group difference in post-intervention PA was small and not statistically significant, and small-cluster robustness checks were consistent. This pattern aligns with meta-analytic evidence that school-based PA interventions often yield null or small effects on MVPA (32–34). Taken together, these findings suggest that, in a single-school, class-based implementation, the additional behavioral gain attributable to the collaborative program may be modest, and may be partly offset by concurrent improvements in control classes. Accordingly, we prioritize adjusted between-group estimates over within-group pre–post changes, which may reflect maturation or school-wide influences affecting both groups.

Several contextual and methodological factors may help explain this pattern. First, PA is sensitive to school-wide scheduling, seasonal variation, and shared environmental changes, which can raise activity levels across classes and reduce detectable contrasts (33, 35). Second, because intervention and control classes were located within the same school, spillover through peer interaction or informal information sharing may have attenuated between-group differences (18, 35). Third, reliance on self-reported PA may limit sensitivity to modest behavioral changes and introduce recall or social desirability bias relative to device-based measures (31, 34).

Notably, the intervention produced clearer improvements in exercise-related cognitions (and perceived benefits), which may represent a proximal change that does not necessarily translate into a large short-term differential increase in PA without stronger structural supports (e.g., protected MVPA time and sufficient dose/intensity) (16, 31). From an implementation perspective, future refinements may therefore strengthen components most directly linked to MVPA opportunities (dose, frequency, and intensity) while maintaining the multi-setting alignment that appears beneficial for shaping exercise-related appraisals.

### 4.2 Impact of the family–school–community collaborative intervention on primary school students’ exercise cognition

The one-academic-year family–school–community collaborative intervention was associated with higher post-intervention exercise cognition, one of the co-primary outcomes. After adjustment for baseline exercise cognition and sex and accounting for class-level clustering, the intervention group scored higher than the control group; this pattern was supported by small-cluster–robust inference, strengthening confidence in the result given the 12-class design. Similar improvements in exercise-related knowledge, attitudes, or intentions have been reported in multicomponent, school-anchored interventions integrating educational and social-support elements for children and parents (16).

These findings suggest that coordinated, multi-setting support may be particularly effective for shaping children’s exercise-related appraisals—especially perceived benefits and barriers—and broader attitudes toward being active (36, 37). Consistent with social-cognitive perspectives, the program may have increased perceived value, understanding, and salience of facilitators/constraints through repeated cues and reinforcement across settings (38). This interpretation aligns with the intervention design: the school component provided structured opportunities and supportive norms, the family component (e.g., parent workshops and sports homework) reinforced routines at home, and community activities expanded access and visibility of opportunities. Reviews similarly emphasize the value of aligning school, family, and community resources to support youth behavior change (16, 19).

Exercise benefits (a secondary outcome) also improved under the class-clustered ANCOVA framework, with a consistent pattern in small-cluster sensitivity checks. Overall, effects were more consistent for cognitive/attitudinal constructs than for PA behavior, which is plausible in school-aged populations: shifts in appraisals may occur earlier and may precede sustained behavioral change (39), whereas detectable differences in daily PA may require greater intervention dose, protected MVPA opportunities, and/or longer-term follow-up (40–43).

### 4.3 Impact of the family–school–community collaborative intervention on the relationship between PA levels and exercise cognition

Building on the intervention findings, we examined whether exercise-related cognitions were associated with students’ PA. In class-clustered regression models, higher post-intervention exercise cognition and perceived exercise benefits were positively associated with post-intervention PA, whereas exercise barriers were not significantly associated with PA. Consistent patterns were observed in change-score models: increases in exercise cognition and benefits were associated with increases in PA, while changes in barriers were not. These associations were modest in magnitude and should be interpreted as correlational patterns rather than evidence of directional influence.

The null association for perceived barriers should not be interpreted as evidence that barriers are unimportant. Multi-setting structural and social supports (e.g., access, scheduling, parental co-activity, and community resources) may buffer the practical impact of constraints without necessarily lowering perceived barriers, thereby attenuating the observed barriers–PA association (16, 44–46). Alternatively, sustained engagement across settings may increase children’s awareness/salience when reporting barriers (e.g., time constraints), which could also weaken statistical associations despite improvements in other cognitive constructs.

From an applied perspective, future programs may address barriers through both cognitive and structural pathways—helping children identify and manage perceived constraints while improving feasibility, access, and family/community support. Longitudinal or experimental mediation designs are needed to test directionality and mechanisms linking cognition-related appraisals with PA change.

### 4.4 Strengths, limitations, and future outlook

To the best of our knowledge, this study is among the first to implement a one-academic-year family–school–community collaborative intervention in primary school students. Key strengths include the multi-contextual, ecologically grounded design integrating school opportunities, family engagement, and community support, and the use of validated instruments and standardized implementation procedures. Methodologically, intervention effects were estimated using ANCOVA with class-clustered robust standard errors, with standardized effect sizes (Hedges’ *g*) and small-cluster–robust inference (exact class-level permutation tests and wild cluster bootstrap-t confidence intervals) to strengthen inference with 12 classes.

However, several limitations should be noted. First, the quasi-experimental, single-school design limits causal inference and external validity. Second, because intervention and control classes were within the same school, contamination/spillover through student interaction could not be ruled out and may have attenuated between-group differences. Third, improvements in both groups suggest possible shared school-wide influences, maturation, and seasonal/timetabling variation, which may partly explain the modest incremental effect on PA. Fourth, PA was assessed via self-report, which may be subject to recall and social desirability bias; the lack of objective PA measures (e.g., accelerometry) limits measurement precision. Finally, we did not collect family-level or socioeconomic covariates (e.g., parental education/household SES), precluding assessment of these potential confounders or moderators. The absence of longer-term follow-up also prevents evaluation of sustainability beyond the post-intervention assessment.

Future research should adopt multi-site or school-level cluster designs, incorporate objective PA assessment and key socio-demographic covariates, and include follow-up measurements to test generalizability, mechanisms, and sustainability.

## 5 Conclusion

This study evaluated a one-academic-year family–school–community collaborative physical activity program in a primary school population. The intervention was associated with higher exercise cognition and higher perceived exercise benefits, whereas the adjusted between-group difference in physical activity was small and not statistically significant after accounting for class-level clustering. Overall, these findings suggest that the program may be more effective in improving children’s benefit–barrier appraisals than in producing a clear incremental increase in self-reported physical activity within a single-school implementation. Future studies should use multi-site, school-level designs, incorporate objective PA measures, strengthen implementation dose and fidelity documentation, and include follow-up assessments to examine generalizability and sustainability.

## Data Availability

The raw data supporting the conclusions of this article will be made available by the authors, without undue reservation.
