# Supplementary material for: Effectiveness of a family–school–community collaborative physical activity intervention
Source: Front Public Health. 2026 Feb 27;14:1767961. doi: 10.3389/fpubh.2026.1767961 (PMC12982172; doi:10.3389/fpubh.2026.1767961)
Supplement: Supplementary file 1 [file Table_1.docx]

**Table S1. Exact class-level permutation inference for ANCOVA treatment effects (two-sided)**

| **Outcome** | **Treat estimate, β** | **Exact permutation p** | **Permutations** | **Treated classes** | **Total classes** |
| --- | --- | --- | --- | --- | --- |
| Physical activity | 0.106 | 0.444 | 924 | 6 | 12 |
| Exercise cognition | 3.940 | 0.0108 | 924 | 6 | 12 |
| Exercise benefits | 3.460 | 0.0270 | 924 | 6 | 12 |
| Exercise barriers | 0.553 | 0.381 | 924 | 6 | 12 |

Note. Exact permutation tests were conducted at the class level by enumerating all allocations of 6 treated classes among 12 classes C(12,6)=924. The test statistic was the ANCOVA treatment coefficient (β) from models specified as Outcome_post ~ treat + Outcome_pre + female, using class as the clustering unit. Two-sided permutation p-values are reported.

**Table S2. Wild cluster bootstrap-t confidence intervals (Webb weights) for ANCOVA treatment effects**

| **Outcome** | **Treat estimate, β** | **SE (clustered)** | **Wild bootstrap-t 95% CI (Webb)** | **Replications (B)** | **Clusters** |
| --- | --- | --- | --- | --- | --- |
| Physical activity | 0.106 | 0.131 | −0.222, 0.436 | 9999 | 12 |
| Exercise cognition | 3.940 | 1.302 | 1.134, 6.761 | 9999 | 12 |
| Exercise benefits | 3.460 | 1.300 | 0.394, 6.432 | 9999 | 12 |
| Exercise barriers | 0.553 | 0.585 | −0.781, 1.869 | 9999 | 12 |

Note. SE = standard error; CI = confidence interval. Wild cluster bootstrap-t confidence intervals were obtained using Webb six-point weights with 9,999 replications, appropriate for inference with a small number of clusters. The underlying ANCOVA model was Outcome_post ~ treat + Outcome_pre + female, with class as the clustering unit.

**Table S3. Sensitivity analysis: class-level difference-in-differences (DID) using class mean change scores**

| **Outcome** | **Mean Δ (treated classes)** | **Mean Δ (control classes)** | **DID (treated − control)** | **SE** | **95% CI** | **p** | **Hedges’ g (on class Δ)** |
| --- | --- | --- | --- | --- | --- | --- | --- |
| Physical activity | 0.395 | 0.322 | 0.073 | 0.080 | −0.107, 0.253 | 0.384 | 0.486 |
| Exercise cognition | 3.475 | 2.145 | 1.330 | 2.129 | −3.587, 6.246 | 0.550 | 0.333 |
| Exercise benefits | 2.488 | 0.559 | 1.930 | 2.143 | −2.879, 6.739 | 0.390 | 0.480 |
| Exercise barriers | 0.986 | 1.586 | −0.600 | 1.402 | −3.735, 2.535 | 0.678 | −0.228 |

Note. Δ denotes the class-level mean change score (post − pre). DID represents the between-group difference in mean change at the class level. SE and CI are based on a class-level comparison (6 treated vs 6 control classes); precision is reduced due to aggregation to 12 class-level observations.

**Table S4. Exploratory association analysis (post-intervention levels): PA_post regressed on cognition/benefits/barriers with clustering**

| **Predictor (post)** | **β** | **SE (clustered by class)** | **95% CI** | **p (clustered)** | **N students** | **N classes** |
| --- | --- | --- | --- | --- | --- | --- |
| Exercise cognition_post | 0.00686 | 0.00214 | 0.00266, 0.01106 | 0.001 | 515 | 12 |
| Exercise benefits_post | 0.00851 | 0.00256 | 0.00351, 0.01352 | <0.001 | 515 | 12 |
| Exercise barriers_post | 0.00337 | 0.00449 | −0.00544, 0.01218 | 0.453 | 515 | 12 |

Note. Models were specified as PA_post ~ Predictor_post + PA_pre + treat + female with class-clustered robust SEs (12 classes). SE = standard error; CI = confidence interval. Analyses used complete cases for the variables included in each model (resulting in N = 515). These analyses are exploratory and not intended for causal inference.

**Table S5. Exploratory association analysis (change scores): ΔPA regressed on Δcognition/Δbenefits/Δbarriers with clustering**

| **Predictor (change)** | **β** | **SE (clustered by class)** | **95% CI** | **p (clustered)** | **N students** | **N classes** |
| --- | --- | --- | --- | --- | --- | --- |
| ΔExercise cognition | 0.00488 | 0.00116 | 0.00260, 0.00716 | <0.001 | 515 | 12 |
| ΔExercise benefits | 0.00667 | 0.00140 | 0.00392, 0.00941 | <0.001 | 515 | 12 |
| ΔExercise barriers | −0.00225 | 0.00372 | −0.00954, 0.00503 | 0.544 | 515 | 12 |

Note. Δ denotes individual change (post − pre). Models were specified as ΔPA ~ ΔPredictor + treat + female with class-clustered robust standard errors. These analyses are exploratory.

**Table S6. Intraclass correlation coefficients (ICC) at baseline by class**

| **Outcome** | **ICC (baseline)** | **Estimation method** | **Variance (between classes)** | **Variance (within classes)** | **N (baseline)** | **Classes** |
| --- | --- | --- | --- | --- | --- | --- |
| Physical activity | 0.114 | MixedLM | 0.0471 | 0.3642 | 515 | 12 |
| Exercise cognition | 0.027 | MixedLM | 7.3359 | 262.2718 | 515 | 12 |
| Exercise benefits | 0.0349 | MixedLM | 7.7531 | 214.5772 | 515 | 12 |
| Exercise barriers | 0.0398 | MixedLM | 2.3066 | 55.6670 | 515 | 12 |

Note. ICC = intraclass correlation coefficient estimated at baseline (pre-intervention) using random-intercept mixed-effects models with class as the clustering unit.
